# Management of concomitant strip and apical perforation in mandibular first molar

**DOI:** 10.12669/pjms.38.7.6009

**Published:** 2022

**Authors:** Mustafa Hussein Al Attas, Thiyezen Abdullah Aldhelai, Muhammad Qasim Javed

**Affiliations:** 1 Mustafa Hussein Al Attas, Saudi Board in Endodontics, Assistant Professor, Department of Conservative Dental Sciences and Endodontics, College of Dentistry, Qassim University, Saudi Arabia; 2 Thiyezen Abdullah Aldhelai, PhD, Department of Orthodontic and Pediatric Dentistry, College of Dentistry, Qassim University, Buraydah, Kingdom of Saudi Arabia. Department of Orthodontics and Pediatric Dentistry, Faculty of Dentistry, Ibb University, Ibb, Yemen; 3 Muhammad Qasim Javed, FCPS. Assistant Professor, Department of Conservative Dental Sciences and Endodontics, College of Dentistry, Qassim University, Saudi Arabia

**Keywords:** Apical Perforation, Strip perforation, Mineral Trioxide Aggregate

## Abstract

Iatrogenic perforation is a complication that can occur during endodontic treatment. If left untreated, it adversely affects the prognosis of the tooth. The use of optimal magnification and appropriate repair material help in achieving favourable outcome. The current case report illustrates the management of concomitant strip and apical perforation in the mesial canals of lower first molar of 13 years old paediatric patient. The management of perforations was done with MTA under 25x magnification of a dental operating microscope. The periapical radiograph and clinical investigations revealed complete bone formation at the furcal area adjacent to the repaired strip perforation and ossification of the apical lesion indicating a favourable healing outcome at 18 months of follow up.

## INTRODUCTION

Root canal perforation is a passage of communication formed between the root canal system (RCS) and the surrounding periodontium.^1^ The communication passage could be the result of pathological processes such as resorptive activity or carious lesions.^2^ Additionally, iatrogenic mishap by an operator can lead to perforation.^3^,^4^ There are several types of perforations that include furcation perforation, strip perforation, apical perforation and crestal perforation.^4^ The strip perforation occurs at the lateral walls of canal, most commonly on the inside walls of the curved canals in first mandibular molars.^5^ The apical perforation results from apical transportation that represent a deviation from the normal pathway of canal anatomy, whereas, crestal perforation is located at the cervical region and opens into the gingival sulcus.^4^ The adverse consequence of perforation is that it complicates RCS disinfection during chemico-mechanical preparation by hindering the accurate negotiation of the canals. Moreover, it enhances the risk of hypochlorite accident secondary to the extrusion of irrigation solutions.^6^ It also makes the obturation phase more complex by making it difficult to restrict the root canal filling material within the confines of the canals.^1^,^6^ For all the aforementioned reasons, presence of a perforation might have a negative impact on the success rate of root canal treatment and tooth survival.^7^,^8^ The literature has highlighted the perforation size, site and time of repair as the critical factors that dictate the success of perforation repair. The early repair, small size and apical location of the perforation defect will result in favourable outcome.^1^ The materials such as, Amalgam,^9^ Super EBA,^10^ Glass Ionomer^11^ and MTA^12^,^13^ have been used for perforation repair. Several studies have shown superior results when perforations are repaired with MTA.^13^,^14^

The current case report describes the management of concomitant strip perforation and apical perforation in the mesial canals of the lower first molar in a 13 years old child with 18- months follow-up.

## CASE PRESENTATION

**Fig.1:**
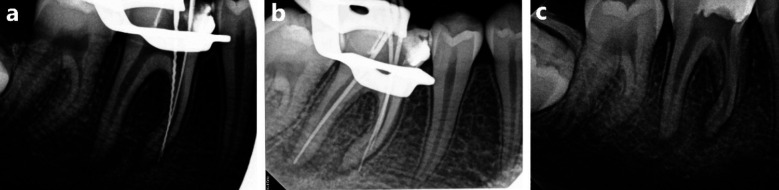
Previous treatment radiographs (a) Showing two files perforating the mesial canals, (b) Showing 2 gutta percha cones perforating the mesial canals, (c) Pre-operative periapical radiograph.

A 13-years-old male patient was referred by the general dental practitioner (GDP) to the university endodontic clinics. Patient presented with a complain of severe pain while eating in the lower right quadrant. The pain started two weeks ago after some complication occurred during endodontic treatment of tooth no 46 that was initiated by GDP. The assessment of treatment notes and intraoral periapical radiographic (IOPAR) records confirmed the presence of concomitant strip perforation and apical perforation with transportation in the mesial canals of tooth 46 (Fig.1a and 1b). The medical history was insignificant. The patient was an irregular dental attendee. The findings of the extraoral examination were insignificant. The intraoral examination showed healthy gingival tissue. Tooth 46 that was temporarily restored was tender to percussion and palpation. The probing pocket depth around the tooth was within normal limits. The new IOPAR showed a widened periodontal ligament space around the mesial root of the tooth at the furcation area with apical radiolucency (Fig.1c). The diagnosis of previously initiated therapy with symptomatic apical periodontitis was made. The patient and his father were informed about the diagnosis and were presented with the possible treatment options with their pros and cons. The possible treatment options included 1) extraction and replacement with a removable partial denture, 2) Root canal treatment of the distal root followed by a resection of mesial root and stainless-steel crown (SSC), and 3) repair of perforations, completion of root canal treatment and SSC. The patient opted for the third option in an attempt to salvage the tooth.

**Fig.2:**
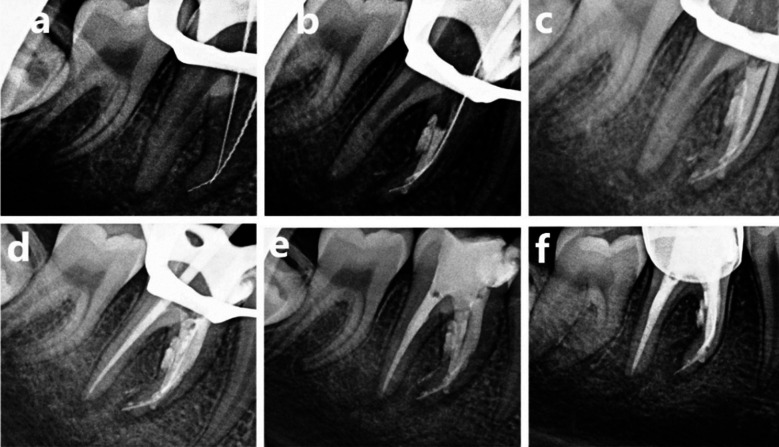
(a) Renegotiation of mesial canals and working length determination, (b) MTA packing for perforation repair and obturation of ML canal, (c) and (d) Master cone gutta percha for obturation of MBC and Distal canal, respectively (e) Coronal restoration by glass ionomer cement, (f) Coronal SSC Coverage.

The inferior alveolar nerve block was administered by injecting 1.8ml of 2% mepivacaine hydrochloride with 18 micrograms of adrenaline (Scandonest, Septodont) as local anaesthetic and rubber dam isolation of tooth 46 was performed. Subsequently, the temporary filling was removed for accessing the canal orifices. The mesial canals were renegotiated under magnification of a dental operating microscope (OPMI Carl Zeiss, Oberkochen, Germany). The pre-curved stainless-steel hand K-files (#10,15,20) were used to follow the original path of canals and achieve the working length of the canals. The working length was confirmed by taking an IOPAR radiograph (Fig.2a). The presence of a strip perforation at the middle third of mesiolingual canal was confirmed by visual examination under a microscope and by using an electronic apex locator (J. Morita Co., Kyoto, Japan). Both mesial canals were apically enlarged to their full lengths by stainless steel K file to size 25 following their natural anatomical pathways with anti-curvature filing technique in combination with irrigation by 3% NaOCl (Parcan, Septodont) and saline. The distal canal was cleaned and shaped to its full length using Protaper NEXT system to size X3 and copiously irrigated by 5.25 % NaOCl solution (Dentaflux). Considering the advantage of mesial canals being joined apically [Vertucci Type-II (2-1 configuration], a stainless-steel K file was inserted in mesio-buccal canal (MBC) to its full length to maintain the original pathway of the canal. Subsequently, the mesio-lingual canal (MLC) was packed with MTA to obturate it, repairing and sealing the strip perforation and the apical perforation, simultaneously (Fig.2b). Following the obturation of MLC, the file placed in the MBC was removed and a gutta percha (GP) cone X2 was inserted into the space maintained by the file. The MB (X2 GP) and distal canals (X3 GP) were obturated using a system B (Sybrondental, Orange, CA, USA) and Hot shot (Discus Dental, Culver City, CA) (Fig.2c and 2d). The tooth was coronally restored by glass ionomer cement and referred to the pedodontics clinic for final coronal coverage with SSC (Fig.2e and 2f).

**Fig.3:**
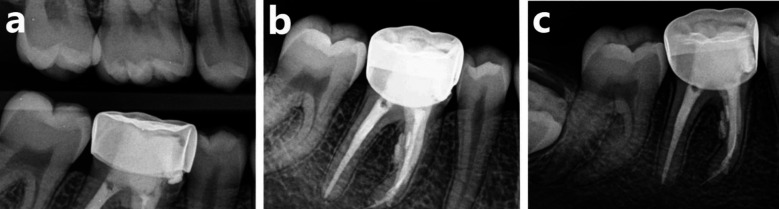
Follow up Radiographs at 18 months (a) Bitewing radiograph, (b) IOPA showing complete furcal healing, (c) IOPA showing complete periapical healing.

The tooth was found to be asymptomatic to percussion and palpation at 18 months follow up. The post-operative radiographs showed fully healed bone at the furcation area adjacent to the perforation site with the normal width of periodontal ligament space, and initial ossification of the apical lesion adjacent to the mesial root, representing a good healing outcome (Fig.3).

## DISCUSSION

The incidence of iatrogenic root perforations during root canal treatment ranges from 2% to 12%.^1^,^7^,^15^ Kvinnsland et al. noted that 53% of the iatrogenic root perforations occur during post space preparation while the remaining 47% take place during endodontic treatment.^3^ The current report has presented two types of iatrogenic perforations that happened simultaneously during root canal treatment and their successful management. The overzealous instrumentation of root canals without following natural canal curvature resulted in strip perforation.^5^ The strip perforation can be prevented by following the anti-curvature filing method while preparing the curved root canal as reported by Abou-Rass et al. and colleagues.^5^ On the other hand, the failure to keep the canal preparation instruments within the confines of the canal space at the full working length led to apical perforation.

Careful examination and early detection and diagnosis are pivotal for proper management and repair of iatrogenic perforations.^1^,^6^,^16^ It has been shown that early management of a perforation improves the outcome and aids in healing by stopping the progression of periodontal damage.^1^ Considering this, the early referral of the patient to endodontist after perforation played a role in the successful outcome. Several methods have been suggested to detect the presence of perforations such as apex locator, multiple angulated periapical radiographs, dental operating microscope and cone beam computed tomography.^6^,^16^-^18^ The current patient reported to the endodontist with angled IOPA radiographs suggesting the perforations that were confirmed and located by utilizing a dental operating microscope and electronic apex locator.

The success of perforation treatment is high if the canals are properly cleaned and the perforations are repaired by a suitable material such as mineral trioxide aggregate (MTA).^14^ In present case, the perforated canals were irrigated by 3% NaOCl for disinfection to avoid the risk of damage to surrounding tissue by extruded solution.^19^ One study showed high success rate of repair by MTA.^14^ The ability of MTA to seal the defect and promote healing by its osteogenic properties to regenerate cementum and stimulate bone formation explains the high success rate.^12^,^20^ The biocompatibility of MTA, its lack of adverse effect and setting mechanism in a wet environment, make it the material of choice for perforation cases where the risk of material extrusion and exposure to moisture exist.^12^ The coronal coverage of root canal treated teeth is a significant factor that enhances the healing outcome.^21^ Thus, the patient was referred to a pedodontics clinic to receive a stainless-steel crown until complete eruption of the adjacent lower second molar.

## CONCLUSION

The early repair of the iatrogenic perforation defects with MTA under high magnification is a viable treatment modality for improving the prognosis of repair. In the current case, complete bone formation at the furcal area adjacent to the repaired strip perforation and ossification of the apical lesion was noted at 18 months follow up indicating a favourable healing outcome.

### Authors Contribution:

**MHA:** Perioperative care, Patient consent, Patient treatment.

**TAA:** Write up, Literature search, Follow up.

**MQJ:** Write up, Proof reading, and is responsible for integrity of research.
